# Soft and Deformable Thermoresponsive Hollow Rod‐Shaped Microgels

**DOI:** 10.1002/smll.202401376

**Published:** 2024-09-10

**Authors:** Fabian Hagemans, Nabanita Hazra, Viktoria D. Lovasz, Alexander J. Awad, Martin Frenken, Andrey Babenyshev, Olli‐Ville Laukkanen, Dominik Braunmiller, Walter Richtering, Jérôme J. Crassous

**Affiliations:** ^1^ Institute of Physical Chemistry RWTH Aachen University Landoltweg 2 DE‐52074 Aachen Germany; ^2^ VTT Technical Research Centre of Finland Ltd Koivurannantie 1 Jyväskylä 40400 Finland

**Keywords:** anisotropic microgels, capillary interactions, hollow microgels, silica rods

## Abstract

Depending on their aspect ratio, rod‐shaped particles exhibit a much richer 2D and 3D phase behavior than their spherical counterparts, with additional nematic and smectic phases accompanied by defined orientational ordering. While the phase diagram of colloidal hard rods is extensively explored, little is known about the influence of softness in such systems, partly due to the absence of appropriate model systems. Additionally, investigating higher volume fractions for long rods is usually complicated because non‐equilibrium dynamical arrest is likely to precede the formation of more defined states. This has motivated us to develop micrometric rod‐like microgels with limited sedimentation that can respond to temperature and reversibly reorganize into defined phases via annealing and seeding procedures. A detailed procedure is presented for synthesizing rod‐shaped hollow poly(*N*‐isopropylacrylamide) microgels using micrometric silica rods as sacrificial templates. Their morphological characterization is conducted through a combination of microscopy and light scattering techniques, evidencing the unconstrained swelling of rod‐shaped hollow microgels compared to core‐shell microgel rods. Different aspects of their assembly in dispersion and at interfaces are further tested to illustrate the opportunities and challenges offered by such systems that combine softness, anisotropy, and thermoresponsivity.

## Graphical abstract

The synthesis and characterization of micrometric rod‐shaped hollow responsive microgels are outlined, along with proof‐of‐concept experiments that emphasize the potential applications of these systems in various self‐assembly processes, both in dispersion and at fluid interfaces.

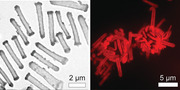

## 1 Introduction

Colloidal rods have a rich phase behavior that strongly depends on their aspect ratio including liquid crystal phases predicted from computer simulations^[^ ^1^, ^2^, ^3^ ^]^ and to some extent observed experimentally.^[^ ^4^ ^]^ These additional liquid crystal phases situated between the liquid and the crystal phases are characterized by long‐range positional and orientational order. Already back in 1949, Onsager showed by theoretical calculations that the isotropic to nematic phase transitions could be purely entropy based for a system based on long repulsive rods.^[^ ^5^ ^]^ Later, computer simulations also indicated the presence of smectic and crystalline phases in the phase diagram.^[^ ^2^, ^6^ ^]^ Experimentally, rod‐shaped particles were first studied at the single particle level by Maeda et al. in 2003.^[^ ^7^ ^]^ Previously, studies mainly rely on many particle properties such as birefringence and scattering.^[^ ^8^, ^9^, ^10^ ^]^ In 2011, Kuijk et al. presented a new colloidal system that allows the study of various aspect ratios, anisotropic enough to form liquid crystal phases.^[^ ^11^ ^]^ The authors extensively investigated the phase behavior of silica rods using small‐angle X‐ray scattering and real space confocal fluorescence techniques.^[^ ^4^, ^12^ ^]^ An experimental phase diagram was constructed including an isotropic, nematic, smectic‐A, and smectic‐B phase depending on the aspect ratio and volume fraction. However, for such hard rods colloids,^[^ ^11^, ^13^ ^]^ it is highly challenging to study phase transitions at very high volume fractions above the glass transition, where the dispersions remain in a metastable state.^[^ ^14^ ^]^

Unlike silica particles, poly(*N*‐isopropylacrylamide) (PNIPAM) microgels are soft^[^ ^15^ ^]^ and responsive to temperature with a volume phase transition temperature (*VPTT*) around 32°C. At high temperatures in their collapsed state, microgels resemble harder colloids whereas in their swollen state at low temperature, they present a much softer interaction potential.^[^ ^16^, ^17^, ^18^ ^]^ The development of rod‐like PNIPAM microgels is therefore expected to open up new opportunities with respect to the microgel responsivity allowing external control of both the dimensions and softness of the rods. In addition, dimensions typically exceeding several hundreds of nanometers are necessary for direct conventional confocal microscopy imaging. However, particularly in the case of inorganic systems such as silica rods that quickly sediment, large dimensions are most of the time accompanied by large buoyant forces. Hollow microgels based on sacrificial template^[^ ^19^, ^20^ ^]^ may thus enable the achievement of large dimensions while maintaining reduced buoyancy. Hollow microgel ellipsoids were realized using silica coated iron oxide spindles as sacrificial templates.^[^ ^21^ ^]^ Latter investigated at oil/water interfaces and supported by computer simulations, it was shown that these microgels may strongly spread at the interface and change their aspect ratio depending on the local thickness of their shell and cavity geometry.^[^ ^22^ ^]^ Nevertheless, because of the small dimensions of their core and their relatively thick microgel shell only low aspect ratios could be achieved and single particles real‐space imaging using confocal microscopy was not possible.^[^ ^21^, ^23^, ^24^ ^]^ Preparing particles from a much larger and more anisotropic core is thus expected to allow for real‐space confocal microscopy imaging of the isotropic to nematic to smectic phase transition. Another strategy to produce highly anisotropic monodisperse microgels consists of the post‐processing of spherical core‐shell microgels into ellipsoids via uniaxial elongation. Aspect ratios up to circa six could be reached.^[^ ^25^, ^26^ ^]^ A similar procedure consists of first post‐processing a functionalized polystyrene core, which is used as a seed for the synthesis of core‐shell microgels.^[^ ^27^ ^]^ In both cases, the strong scattering of the polystyrene core hinders confocal imaging of dense dispersions deep in bulk. Alternatively, anisotropic microgels can be produced via microfluidic techniques^[^ ^28^ ^]^ or projection photolithography.^[^ ^29^ ^]^ The microgels obtained with these techniques are, however, way too large to exhibit significant Brownian diffusion and also quickly sediment. Finally, to conclude this non‐exhaustive list of anisotropic microgels, one alternative namely the use of particle replication in a non‐wetted template (PRINT)^[^ ^30^, ^31^, ^32^ ^]^ could provide some interesting highly defined systems but usually in very limited quantities and with some shape limitations in respect to the molding technique.

Both anisotropic colloids^[^ ^33^, ^34^ ^]^ and microgels^[^ ^16^ ^]^ present interesting interfacial properties motivating, among others, their use as emulsion stabilizers. Besides being highly surface active, similar to some “hard” anisotropic colloids,^[^ ^35^, ^36^, ^37^, ^38^, ^39^, ^40^, ^41^ ^]^ soft anisotropic microgels may experience strong capillary interaction resulting from the complex interplay between their shape and interfacial deformation depending on their architecture, their affinity for the interface and deformability.^[^ ^22^, ^23^, ^27^ ^]^ The development of micrometer‐sized anisotropic hollow microgels thus provides interesting properties to test their interfacial assembly, in particular, considering their eventual ability to bend to adapt to the local curvature. These systems are part of the ongoing development of model responsive rod‐like colloids, such as PNIPAM‐functionalized viruses and self‐assembled DNA‐functionalized rods,^[^ ^42^, ^43^ ^]^ Especially noteworthy are DNA‐linked chains, where the choice of the DNA linker allows for tuning the bending rigidity.^[^ ^44^, ^45^, ^46^ ^]^

In this paper, we present the synthesis of micrometer‐sized PNIPAM based rods and show their potential for studying in situ phase transitions of rod‐shaped model systems. We prepared hollow rod‐shaped microgels using a four‐step synthesis. First, a rod‐shaped template is coated with 3‐(trimethoxysilyl) propyl methacrylate (TPM) by hydrolysis and condensation in the presence of ammonia. Around these particles a layer of fluorescently labeled microgel is grown by a potassium peroxodisulfate (KPS) initiated radical polymerization reaction. Finally, the silica core is removed by etching in a mild sodium hydroxide solution. Transmission electron microscopy (TEM) was employed to characterize the different steps of the synthesis leading to the hollow microgel rods. Stiffness tomography by atomic force microscopy (AFM) in solution and laser scanning confocal microscopy (CLSM) were employed to characterize their properties in the swollen state. Hereby, CLSM measurements were further applied to highlight their assembly in dense dispersions and test their suitability for studying phase transition in situ. Based on their initial characterization from the diverse imaging techniques, their structure and thermoresponsivity were then investigated combining static light scattering (SLS) and dynamic light scattering (DLS). Moving to their interfacial assembly, the spontaneous capillary driven self‐assembly of the rod‐shaped microgels was investigated at planar air/water interfaces. The ability of hollow rod‐shaped microgels to assemble and deform at emulsion droplets is finally tested to illustrate their ability to bend and adapt their shape to the local curvature. The proposed approach thus offers the possibility to directly probe the dynamics and self‐assembly of such rod‐like systems due to their relatively large dimensions and slow dynamics, with limited buoyancy related to their core‐shell architecture, without requiring density matching.^[^ ^47^ ^]^ Additionally, the deformability of the rods is expected to be tuned by the dimensions of the silica template and the elasticity and thickness of the microgel shell.

## 2 Results and Discussion

**Figure 1:**
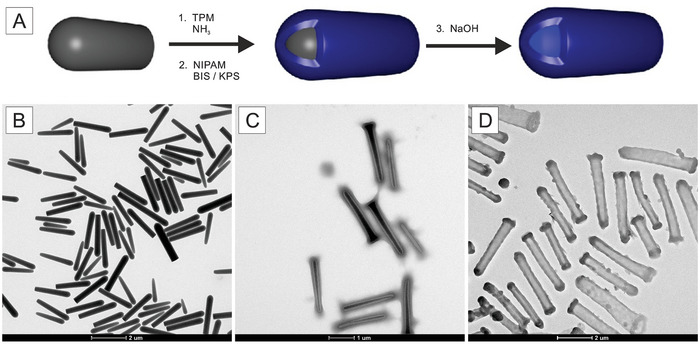
A) Schematic representation of the synthetic procedure to obtain hollow microgels using silica rods as sacrificial templates. B,D) Transmission electron microscopy (TEM) images of B) silica rods, C) microgel‐coated silica rods, and D) etched hollow microgel rods. With respect to their anisotropy, anisotropic microgels tend to assemble side by side into short strings due to capillary interactions.

The microgel rods were synthesized using a four‐step template‐based synthesis (**Figure** **1**A), as described in more detail in the Supporting Information. First, silica rods were synthesized and characterized by transmission electron microscopy (TEM). The particles have a typical “bullet” shape analogous to a spherocylinder truncated at one end, as described in previous studies.^[^ ^11^ ^]^ To reduce their size distribution, the rod dispersion was fractionated by centrifugation to obtain a relatively monodisperse rod batch with an average length, *L*_*C*_ = 2276 ± 313 nm, width, *D*_*C*_ = 313 ± 69 nm, and aspect ratio, ρ_*c*_ = 7.4 ± 0.7 (Figure 1B). The silica rods were then functionalized with vinyl groups through base‐catalyzed hydrolysis and condensation of TPM onto the surface of the silica rods. In the second step, the microgel is grown around the particles during a seeded radical polymerization reaction, with the vinyl groups of the TPM acting as anchors for the polymerization of the microgel. The microgel is cross‐linked using *N*, *N*′‐methylenebis acrylamide (BIS) at a crosslinking density of 7.5 mol.%. The silica template is then removed by etching the particles in a mild sodium hydroxide solution. Both core‐shell and hollow structures were purified by repeated centrifugation and redispersed in pure water.

The successful coating of the silica template with PNIPAM microgel was confirmed by TEM analysis (Figure 1C). After the formation of the microgel shell, the silica core adopted a slightly conical shape as a consequence of mild etching in water at elevated temperatures. This can be attributed to the gradient in the degree of condensation found for these types of rods.^[^ ^48^ ^]^ The etching apparently occurred at a later stage during the reaction, as the cavity seems to have maintained its bullet shape. In Figure 1D, a typical TEM image is shown highlighting the successful dissolution of the silica template. The etched microgel rods generally maintained their bullet shape on the template. The averaged length *L*_*TEM*_ and width *D*_*TEM*_ were evaluated from statistical analysis of the TEM micrographs. The same measurements performed on the core‐shell resulted in *L*_*TEM*_ = 2467 ± 236 nm and *D*_*TEM*_ = 436 ± 53 nm, corresponding to an aspect ratio of 5.7 ± 0.4. Dissolving the core then results in significant flattening of the microgels, characterized by much larger dimensions: *L*_*TEM*_ = 5177 ± 555 nm and *D*_*TEM*_ = 854 ± 116 nm, corresponding to an aspect ratio of 6.1 ± 0.8.

Additionally, the TEM micrographs evidenced increased microgel density at both tips and tails of the particles. A closer inspection reveals higher density at the rim of the flat end of the rods. The growth of the microgel shell onto the surface depends strongly on TPM assembly at the surface of the silica rods. During coating with TPM, higher packing can be achieved in curved areas due to decreased steric hindrance at rounded segments of the particles. Due to curvature on both hemispherical tips and edges of flat circular bases, more TPM was able to bind to silica, explaining the locally denser microgel coating.

In order to gain further insights into swollen microgels, atomic force microscopy (AFM) measurements were performed in solution as described in Supporting Information. The microgels were adsorbed under highly dilute conditions onto a glass slide that had been sonicated for 15 min in isopropanol. This preparation allows for immobilizing microgels on the glass surface. Subsequently, stiffness tomography measurements were performed on a few representative core‐shell and hollow microgels, as shown in Figure S1 (Supporting Information). These experiments indicate that both systems strongly deform at the substrate, as evidenced by their lateral spreading on the order of 800 nm—exceeding their height slightly below 600 nm at midsection. The length of hollow rods was determined to be around 4 μm, significantly larger than that for core‐shell structures, which measured around 3 μm. Interestingly, this length remained lower for hollow microgels than what was observed in TEM experiments. The bullet shape is also preserved in their swollen state, demonstrating that these particles may differ from an ideal rod shape. Remarkably, hollow microgels display very soft behavior due to their cavity; high contact stiffness could not be achieved even with penetration depths exceeding several hundred nanometers. However, as expected, recorded stiffness is far higher at both extremities.

**Figure 2:**
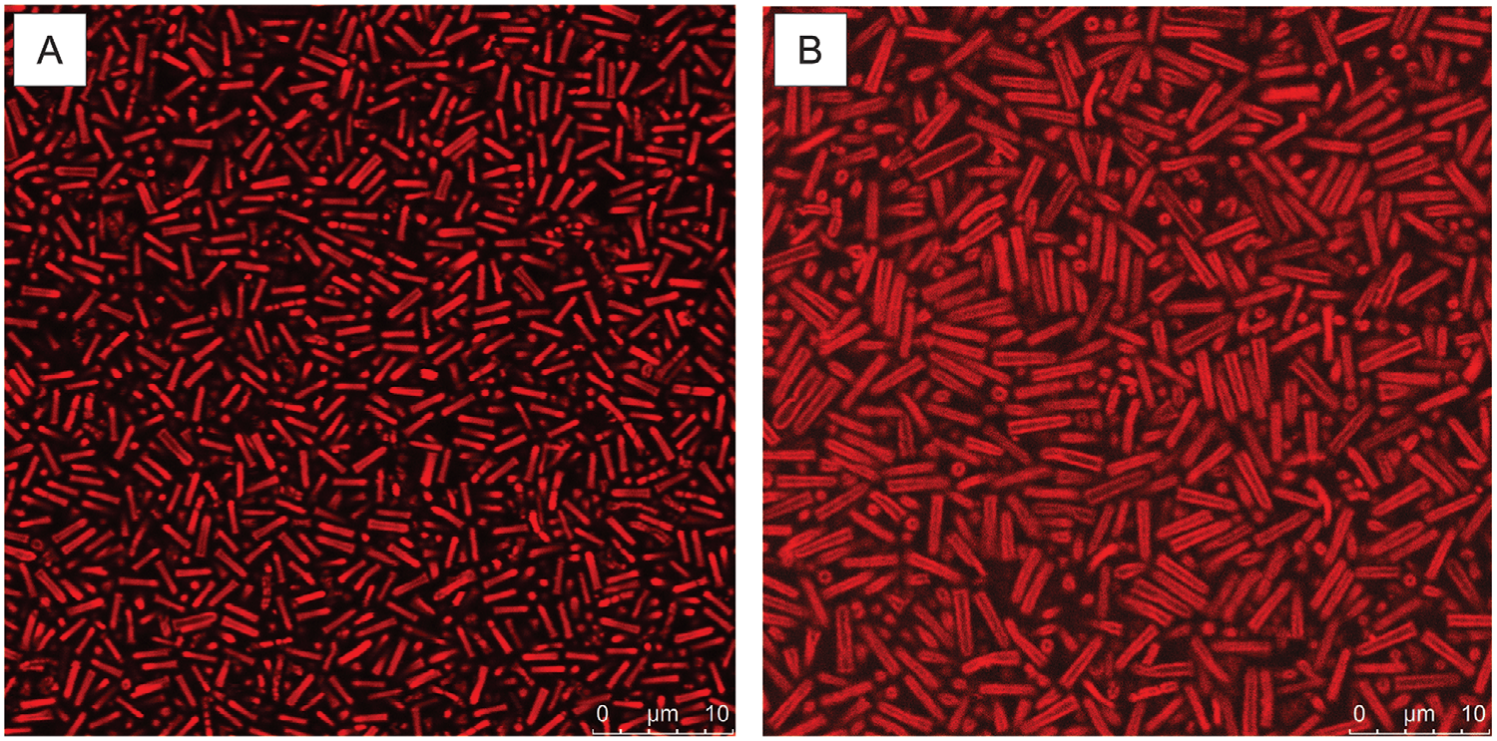
Confocal laser scanning microscopy micrographs of a concentrated suspension of core‐shell (A) and hollow (B) rod‐shaped microgels, imaged at the surface of the coverslip.

Both systems were directly imaged under concentrated conditions via confocal laser scanning microscopy (CLSM) at room temperature, approximately 23 °C. Dilute microgel dispersions were centrifuged at 2000 rpm for 15 min in an Eppendorf tube. After removing most of the supernatant and redispersing the pellet via vortexing and 30 min of sonication, the obtained dispersion was transferred to a confocal preparation where it was sandwiched between a glass slide and a coverslip separated by a watertight 120 μm thick spacer. The corresponding dispersion concentrations were estimated to be 11.8 and 8.8 wt.%. To limit adhesion to the glass substrate, the coverslip was ozone‐treated, and the samples were allowed to relax at 20 °C for 48 h (see Supporting Information for further details on sample preparation). Micrographs captured at the glass coverslip at the same magnification are shown in **Figure** **2**. The samples were found to be in an arrested state, allowing for clear imaging of their structure in suspension. However, their slight rattling against the glass, shown in Videos S1 and S2 (Supporting Information), confirmed that the coverslip treatment prevented irreversible adhesion to the surface.The first striking observation is the large difference in dimensions between the two systems, which was already hinted at by previous TEM characterization. The slow dynamics of the systems allow us to accurately estimate the dimensions of the particles lying within the imaging plane. Such analysis leads to long and short axes estimated at *L*_*CLSM*_ = 2582 ± 230 nm and *D*_*CLSM*_ = 493 ± 71 nm, for core‐shell microgels, and *L*_*CLSM*_ = 4233 ± 383 nm and *D*_*CLSM*_ = 809 ± 14 nm, for hollow microgels. For both systems, this corresponds to an average aspect ratio on the order of 5.3. It is important to keep in mind that such estimates do not account for the full hydrodynamic dimensions of the microgels, which appear as well‐separated entities despite their dynamics being arrested. This further emphasizes the contribution of dangling chains and extended fuzziness of the microgels to their dynamics. We further note that some core‐shell structures appear fragmented as a consequence of partial hydrolysis of the silica core. Additionally, core‐shell microgels are much more fluorescent than hollow microgels, which could relate to a lower swelling degree of their shell, their dielectric environment, or the presence of silica cores. It is also unclear how dissolution of the core under alkaline conditions may have affected shell fluorescence. Interestingly, although the cavity of hollow microgels is clearly visible, no significant bending fluctuations could be observed (see Video S2, Supporting Information). Especially for hollow microgels, we notice that their bullet shape is preserved, reflected in their organization within dense dispersions. Both systems exhibited local nematic ordering at the surface of the coverslip characterized by side‐to‐side organization into small particle stacks. No long‐range ordering could be observed; this is mainly attributed to polydispersity in size and shape among these systems. Deeper in bulk, some microgel rattling was observed; however, both dispersions were found to be arrested (see Videos S3 and S4, Supporting Information). No clear positional or orientational order was recorded, indicating that both dispersions were in a disordered glassy state. By raising the temperature to 31 °C, both systems transitioned into a fluid state due to deswelling of PNIPAM microgel shells as evidenced for hollow microgels in Video S5 (Supporting Information).

**Figure 3:**
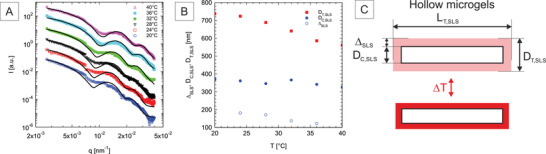
Characterization of the hollow rod‐like microgels from SLS. A) SLS form factors determined at two different wavelengths (symbols) fitted to a polydisperse core‐shell cylinder model (solid lines) from 20 to 40 °C. The different curves have been shifted by a constant factor for clarity. B) Evolution of the cavity diameter, *D*_*C*, *SLS*_, shell thickness, Δ_*SLS*_, and total diameter, *D*_*T*, *SLS*_ = *D*_*C*, *SLS*_ + 2Δ_*SLS*_, determined from the fits (see text for more details). C) Schematic representation of the different average dimensions of the hollow rod‐like microgels at 20 and 40 °C, drawn to scale.

In order to gain further insights into the structure of the core‐shell and hollow microgels, SLS experiments were performed at different temperatures under dilute conditions. The measurements were conducted on a custom‐designed SLS setup with two different lasers emitting at 407 and 819 nm, respectively. The two measurements were then rescaled to overlap in the intermediate *q*‐region (see **Figure** **3**A). The different scattering curves corresponding to the form factor of the hollow microgels exhibit interesting features such as minima and maxima characteristic of different length scales. We observed significant smearing around the first minimum, which is particularly prominent at λ = 819 nm; one underlying reason could be related to shape fluctuations and bending of the hollow rods. As the temperature increases, the curves become more structured, and the minima shift to higher *q*‐values, indicating a reduction in the dimensions of the microgels. The combined measurements at the two wavelengths—excluding the highly smeared first minimum region measured at 819 nm—were analyzed using a core‐shell cylinder model^[^ ^49^ ^]^ (see solid line in Figure 3A). This model was selected for its robustness and simplicity in demonstrating both hollowness and thermoresponsivity of the microgels, characterized by cavity diameter *D*_*C*, *SLS*_, shell thickness Δ_*SLS*_, total diameter *D*_*T*, *SLS*_, and total length *L*_*T*, *SLS*_ = *L*_*C*_ + 2 · Δ_*SLS*_. A constant polydispersity of 15%, assuming a Gaussian distribution, was considered for both core diameter and shell thickness. A schematic representation of the core‐shell cylinder model along with its characteristic dimensions is shown in Figure 3C. It is important to note that since the long axis is much greater than the lateral dimensions, this parameter does not significantly influence the fitting outcome. This simple approach captures the main features of the scattering curves effectively. The description using the core‐shell cylinder model is particularly accurate above the *VPTT*, where a more homogeneous shell density is expected. At lower temperatures, our model does not account for shell fuzziness, which could explain some discrepancies between experimental results and fits. The analysis of fit parameters shown in Figure 3B confirmed that the microgels remain hollow, with *D*_*C*, *SLS*_ decreasing from 370 to 326 nm, which aligns with core diameter determined by TEM. *D*_*T*, *SLS*_ shows a more marked transition from 738 to 562 nm, with a transition occurring around 32 °C. Accordingly, Δ_*SLS*_ decreased from 184 to 118 nm. The corresponding configurations at 20 and 40 °C derived from the model are shown in Figure 3C. We further note that *D*_*T*, *SLS*_ is similar but slightly lower than that the total width determined from confocal measurements. The difference observed between these two methods at lower temperatures can be attributed to microgel fuzziness not accounted for in our model. Due to the large difference in refractive index between silica cores and microgel shells, we investigated core‐shell microgel rods under index‐matching conditions using a 97 vol% DMSO/water mixture (see Figure S2, Supporting Information) at 20 °C following similar procedures. Values for *D*_*C*, *SLS*_ and *D*_*T*, *SLS*_ were determined to be 263 and 648 nm, respectively. The value of *D*_*C*, *SLS*_ is lower than that obtained from TEM measurements of silica cores; we relate this discrepancy to partial hydrolysis of those cores. Additionally, *D*_*T*, *SLS*_ was found to be slightly larger than that observed in confocal measurements, which may partly result from differing solvent conditions.

**Figure 4:**
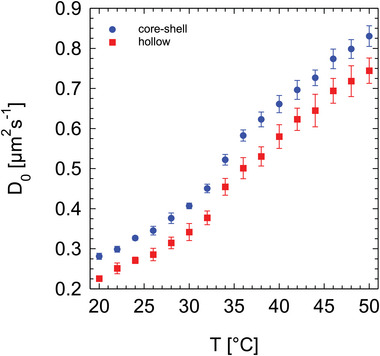
Evolution of the diffusion coefficient as a function of temperature, measured for dilute core‐shell (filled squares) and hollow (filled dots) rod‐shaped microgels. The error bars represent the standard deviation of the diffusion coefficient measured at different scattering angles.

We proceeded with the characterization of their diffusion and hydrodynamic properties. The length and width of the microgel rods, as well as their responsivity to temperature, were estimated by measuring their diffusion coefficient, *D*_0_, using dynamic light scattering, as shown in **Figure** **4**. We observed that the two systems exhibit similar behavior, characterized by an inflection point around the volume phase transition of PNIPAM microgels between 32 and 35 °C, confirming their thermoresponsivity. Additionally, we noted that the rod‐like core‐shell microgels diffuse systematically faster than the hollow ones. This observation aligns with previous confocal analysis, which indicated significantly lower dimensions for core‐shell microgels compared to hollow ones. The error bars represent the standard deviation of measurements performed at different scattering angles. Furthermore, the average polydispersity index (PDI) from the second cumulant analysis was determined at 20 °C to be 0.2 ± 0.1 for core‐shell microgels and 0.3 ± 0.1 for hollow microgels, which is consistent with the polydispersity determined from TEM analysis.

**Figure 5:**
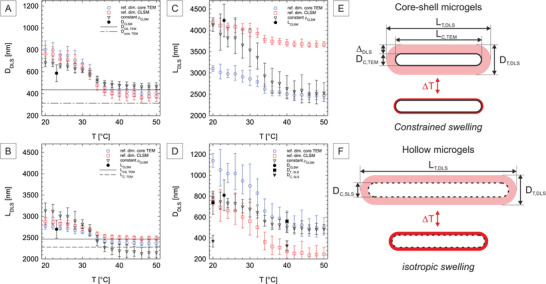
Characterization of the hydrodynamic properties of core‐shell and hollow rod‐like microgels from DLS measurements as a function of temperature, considering a spherocylinder model and the experimental diffusion coefficients. A, B) Estimation of the hydrodynamic diameter, *D*_*T*, *DLS*_, and length, *L*_, *DLS*_, of core‐shell rod‐shaped microgels. C, D) *D*_*T*, *DLS*_ and *L*_, *DLS*_ of hollow rod‐shaped microgels. The different models use either the dimensions of the core determined from TEM (ref. dim. TEM core), dimensions from CLSM (ref. dim. CLSM), or a constant aspect ratio determined from CLSM analysis (constant ρ_*CLSM*_) as references (see text for more details). These estimates are further compared to dimensions determined by previous characterization techniques. E, F) Schematic representations of the different average dimensions at 20 and above 40 °C, drawn to scale for both systems. The core‐shell microgels are described using dimensions determined with the silica core as reference, indicating swelling constrained by the presence of the core, whereas the assumption of a constant aspect ratio characteristic of isotropic swelling seems more appropriate for hollow microgels.

Based on the measurements of *D*_0_ and assuming a spherocylinder model detailed in the Supporting Information,^[^ ^50^, ^51^ ^]^ we attempted to determine the hydrodynamic diameter *D*_*DLS*_ and length *L*_*DLS*_ of core‐shell rod‐shaped (**Figure** **5**A,C) and hollow (Figure 5B,D) microgels. Such analysis strongly depends on the choice of the reference system. Our initial model considers a spherocylinder with the average dimensions of the silica template, *L*_*C*_ and *D*_*C*_. Similar to our previous study on the PNIPAM functionalization of anisotropic mesoporous silica particles,^[^ ^52^ ^]^ the first model (ref. dim. core TEM) assumes that the microgel shell has a constant thickness, Δ_*DLS*_, at each temperature. The total hydrodynamic length *L*_*DLS*_ and diameter *D*_*DLS*_ were then derived by adjusting Δ_*DLS*_ to match the experimental value measured for *D*_0_ at different temperatures. The second model (ref. dim. CLSM) follows a similar approach, using average dimensions determined by CLSM as references. Both models thus refer to constrained swelling of the microgel shell. Finally, the third model (constant ρ_*CLSM*_) assumes that the aspect ratio of the rods remains constant through the volume phase transition, defined by ρ_*CLSM*_. The obtained dimensions are further compared to those determined by previous characterization techniques, summarized in Table S1 (Supporting Information). For core‐shell microgels, these three different approaches initially appear relatively equivalent; in particular, the first and second models provide almost similar results that agree with other techniques. From this analysis, we concluded that swelling of core‐shell systems is most likely constrained by the silica core. Using this silica core as a reference, *L*_*T*, *DLS*_ varies from approximately 2768 to 2385 nm and *D*_*T*, *DLS*_ from 805 to 422 nm between 20 and 40 °C. The characteristic dimensions of this model in both swollen and collapsed states are schematically displayed in Figure 5E. This suggests that not only can the total volume of particles *V* be adjusted with temperature, but also their aspect ratio, as illustrated in Figure S3 (Supporting Information). The swelling ratio, defined as α = *V*(*T*)/*V*_*collapsed*_, was estimated at approximately 4.3 at 20 °C, while ρ_*DLS*_ varied from about 3.4 to 5.6 between 20 and 40 °C. In contrast, for hollow microgels, these three different models yield dramatically different results. The evolution of corresponding aspect ratios and swelling is further illustrated in Figures S3C,D (Supporting Information). Unlike core‐shell systems, only the third approach considering a constant aspect ratio ρ_*CLSM*_ aligns with other techniques; specifically, this model accurately reproduces system dimensions measured by CLSM and SLS. The characteristic dimensions for this model in both swollen and collapsed states are schematically displayed in Figure 5F. This analysis suggests that hollow microgels swell isotropically after dissolution of their cores while maintaining their shape with temperature changes. Such isotropic/unconstrained swelling has been observed in other anisotropic PNIPAM‐based microparticles synthesized via molding techniques–specifically particle replication in a non‐wetting template (PRINT)^[^ ^31^ ^]^ or projection photolithography.^[^ ^29^ ^]^ In this case, *L*_*T*, *DLS*_ varies from approximately 4127 to 2637 nm and *D*_*T*, *DLS*_ from 789 to 504 nm between 20 and 40 °C. Their swelling ratio α was estimated around 4.2 at 20 °C, which is nearly identical to that observed for core‐shell systems. Notably, hollow microgels seem to expand significantly after dissolution of their cores and exhibit cavities exceeding those of sacrificial silica templates–a phenomenon previously reported for large spherical microgel capsules using micrometer‐sized silica as sacrificial templates with relatively thin shells compared to their cores.^[^ ^20^, ^53^ ^]^ This effect is likely related directly to release constraints on the microgel shell as well as grafting density within it; however, it may also depend on system dimensions and shell‐to‐core size ratios. Indeed, smaller hollow microgels with thicker shells compared to their core radii have also been shown to expand toward the cavity.^[^ ^19^, ^54^ ^]^ A more systematic investigation supported by molecular dynamics (MD)^[^ ^48^ ^]^ or dissipative particle dynamics (DPD)^[^ ^23^ ^]^ computer simulations should be able to address this question. Furthermore, the fact that the hollow systems maintain an anisotropic shape suggests that their elastic energy greatly exceeds the effect of surface tension, which clearly depends on the dimensions of the systems.^[^ ^15^ ^]^

Due to their interfacial activity, PNIPAM microgels have been found to lower interfacial tension when adsorbed at air/water or oil/water interfaces^[^ ^55^, ^56^ ^]^ and can be employed as responsive emulsion stabilizers, where the emulsion can be broken above the *VPTT*.^[^ ^57^, ^58^, ^59^ ^]^ Additionally, interfacially active large rod‐like colloidal particles are known to self‐assemble at interfaces due to capillary interactions created by interfacial deformation.^[^ ^33^, ^60^, ^61^ ^]^ Soft anisotropic microgel systems are far less studied at the interface but offer interesting properties, as they can strongly deform depending on their softness and architecture while still exhibiting capillary interactions.^[^ ^23^, ^24^, ^27^ ^]^

**Figure 6:**
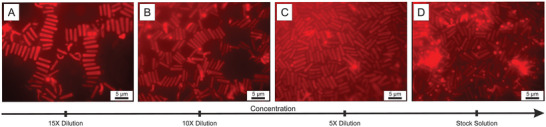
Fluorescence micrographs of the air‐water interfacial assembly of hollow rod‐shaped microgels measured at different concentrations, with the stock dispersion concentration being on the order of 0.1 wt.%. Scale bars: 5 μm.

The self‐assembly of the hollow rod‐shaped microgels was therefore tested at the air‐water interface. A customized magnetically sealed cell was employed to directly image the microgels at the interface using fluorescence microscopy (see Supporting Information for further details). A thin liquid film is created within the cell that is hermetically sealed, preventing the complete evaporation of the dispersion. This setup enables us to image an almost completely flat interface in the middle of the cell using an inverted fluorescence microscope equipped with a high‐magnification immersion objective, as described in the Supporting Information (Figure S5, Supporting Information). Hereby, the spontaneous adsorption and assembly of the hollow rod microgels from the dispersion at the interface could be directly monitored. The interfacial assembly was investigated at various surface coverages by adjusting the dispersion concentration. Besides being highly surface‐active, the hollow microgel rods were found to significantly flatten and spread at the interface. We attribute this to the documented surface activity of PNIPAM microgels and their ability to reduce surface tension by adsorbing at the air/water interface.^[^ ^15^ ^]^ Furthermore, the hollow microgels are more deformable due to their cavity,^[^ ^20^, ^62^ ^]^ which favors their spreading at the interface to maximize their contact area. Their average diameter and length were measured at the interface under maximum dilution, yielding *L*_*int*_ = 4822 ± 529 nm and *d*_*int*_ = 1000 ± 175 nm. Interestingly, although they deform significantly at the interface, we found that the microgel rods assemble into long arrays at low surface coverage, consisting of rods lying side by side (see **Figure** **6**A,B), as expected for “hard” capillary assembly of rods with large aspect ratios.^[^ ^33^, ^60^, ^61^ ^]^ Therefore, we can conclude that they significantly deform the interface. The average side‐to‐side distance within these stacks was estimated at *d*_*SS*_ = 1612 ± 88 nm. Similar observations were made for core‐shell microgels, which were investigated under dilute conditions at a concentration of approximately 0.1 wt.% (see Figure S5, Supporting Information). However, these particles appeared less surface‐active and did not exhibit such long‐range ordering as observed in hollow systems. Additionally, this system tends to sediment much more quickly, reducing the number of particles present at the air/water interface. Still, it was observed that a side‐to‐side interfacial assembly was preferred. Locally analyzing the center‐to‐center distance of particles present in such assemblies led to *d*_*SS*_ = 1229 ± 115 nm. Again, this analysis confirmed that hollow microgels are far more deformable than their core‐shell counterparts; indeed, grafting chains to a solid core hinders lateral spreading. This is illustrated by the ratio *d*_*SS*_/*D*_*DLS*_, which characterizes each type's ability to deform at interfaces–determined to be 2.0 for hollow microgels and 1.5 for core‐shell systems. The different dimensions of both systems measured in bulk and at interfaces are summarized in Table S1 (Supporting Information). We found it remarkable that this distance was, first, much larger than any of the observed dimensions of the system and, second, extremely well‐defined and independent of the number of microgels in the stacks, even though the microgels clearly differ in their dimensions and shapes. The nature of such long‐range interactions has not yet been elucidated and could be related to either a thin, highly spread microgel corona or additional electrostatic interactions. Increasing the microgel concentration further results in a denser assembly with local nematic regions. Ultimately, at the highest concentration, a less defined assembly is observed, comprising several microgels adsorbed at the interface by one of their tips. Keeping in mind the “bullet” shape stemming from the silica template, we may also expect different adsorption rates at the two tips since one is flat and the other is rounded.^[^ ^63^ ^]^ Further investigations into the adsorption pathway may enable control over the proportion of microgels adsorbing parallel to versus orthogonal to the interface.^[^ ^63^ ^]^

**Figure 7:**
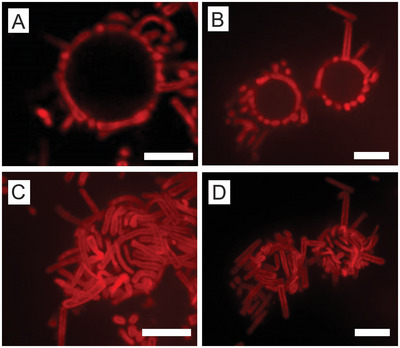
Hollow rod‐shaped microgels assembled at (curved) oil‐water interfaces. A,B) Typical oil droplets packed with microgel rods after emulsification. C,D) *z*‐projections of the droplets shown in (A‐B). Scale bars: 5 μm.

To gain further insights into this special feature and the ability of hollow microgel rods to deform at the interface, their adsorption at the surface of small decane emulsion droplets was investigated. The emulsification process was performed in the presence of the microgels, which served as stabilizing agents to create so‐called “mickering” emulsions.^[^ ^16^, ^17^, ^64^ ^]^ These emulsions were imaged via confocal microscopy, as shown in Figure 7. The 2D equatorial cross‐sections of these droplets in Figure 7A,B demonstrate the dense packing of microgels at the interface, primarily along the interface, as well as their ability to adapt to local curvature. Additionally, secondary adsorption at the tip could be imaged, where the microgels exclusively adsorbed with their flat ends at the interface. These observations were supported by 3D reconstructions of the same emulsion droplets based on maximum intensity projections acquired from *z*‐stacks of the lower half of the emulsion droplets (see Figure 7C,D). The 3D reconstructions further allow us to clearly highlight that some rods are significantly deformed to maximize their packing at the interface (**Figure** **7**).

## 3 Conclusion

In our proof‐of‐concept experiments, we have demonstrated that silica rods, which serve as benchmarks for studying the phase diagram of rods in real space,^[^ ^4^, ^12^ ^]^ can be functionalized with a microgel shell. The silica core can be used as a sacrificial template to obtain thermo‐responsive hollow microgels with significantly reduced buoyancy. These new systems thus open avenues for studying complex phase behavior and the influence of softness in rod‐like particles. Interestingly, the microgels were found to become more anisotropic after the dissolution of the silica core, which should be supported in future studies by computer simulations following the methodology presented in earlier research.^[^ ^23^ ^]^ Our analysis further demonstrates that the rod‐shaped hollow microgels swell isotropically, whereas the swelling of the core‐shell microgels is constrained by the presence of the core. Accordingly, the effective volume fraction in dispersion and particle softness can be conveniently adjusted with temperature; in the case of the core‐shell system, anisotropy can also be tuned with temperature. The thermoresponsivity of these systems may therefore allow for thermal annealing procedures to trigger and promote defined liquid crystalline phases or to investigate their colloidal glass transition. Moreover, such particles are highly surface‐active and assemble at air‐water interfaces via defined capillary interactions. At emulsion droplets, hollow rod‐like microgels can bend to adapt to local curvature. Future investigations may focus on their deformation and assembly either in dispersion at high volume fractions or at fluid interfaces under controlled uniaxial compression.

It is important to note that the synthetic procedure is highly versatile; the original silica rods can be overgrown with different aspect ratios,^[^ ^11^ ^]^ and the microgel shell can be synthesized with varying cross‐linking densities, thicknesses, and functional moieties. Rod‐like microgels could thus be obtained with diverse architectures, dimensions, anisotropies, softness levels, bending rigidities, and functionalities. This versatility should enable a vast range of applications–from serving as model systems to probe thermotropic phase behavior or study interfacial assembly to acting as emulsion stabilizing agents. Furthermore, subsequent batch procedures based on sacrificial templates may facilitate the creation of anisotropic microgels in larger quantities suitable for tissue engineering applications where functional anisotropic microgels have become prominent due to their ability to pack into non‐closed structures or direct cellular growth when aligned in a gel.^[^ ^32^, ^65^ ^]^

## 4 Experimental Section

### Materials

*N*‐isopropylacrylamide (NIPAM; Sigma–Aldrich), *N*, *N*′‐methylenebis acrylamide (BIS; Sigma‐Aldrich), Potassium peroxodisulfate (KPS 99%; Across Organics), Methacryloxyethyl thiocarbamoyl rhodamine B (MRB‐dye; Sigma‐Aldrich), Tetraethyl orthosilicate (TEOS; Sigma–Aldrich), 3‐(trimethoxysilyl) propyl methacrylate (TPM; Sigma–Aldrich), Ammonia (26.1 %; Merck), polyvinylpyrrolidone (PVP; Sigma–Aldrich, *Mn* = 40 kgmol^‐1^), Ethanol absolute (VWR) and sodium hydroxide (0.1M NaOH; VWR) were used as received. MilliQ water was used for performing the synthesis and experiments.

### Microgel Synthesis

The particles were synthesized using a four‐step process. In the first step, a silica template consisting of silica rods was prepared. Then, in the second step, the surface of these particles was modified with vinyl groups to ensure the attachment of the microgel layer in the next step. In the fourth and final step, the silica template was removed by mild etching in sodium hydroxide. A detailed description of the synthesis was provided in the supporting information.

### Methods

Transmission electron microscopy (TEM) measurements were performed using a Philips Tecnai F20 instrument operated at 200 kV. Samples were prepared by drying a 10 μL droplet on a formvar‐carbon coated 3.05 mm Plano‐EM 200 mesh copper grid.

Atomic force microscopy (AFM) measurements were performed on a Dimension Icon AFM with a closed loop (Veeco Instruments Inc., software Nanoscope 9.4, Bruker Corporation). First, glass slides measuring 2.2 × 2.2 cm (Menzel‐Gläser, n4) were cleaned by ultrasonication in isopropanol for 15 min. Then, the microgel solution was applied to a glass slide and left for 1 h to allow for adsorption. Subsequently, the slide was rinsed several times with 2 mL of water to remove any unabsorbed microgels and was measured at 27 °C in water. The 3D stiffness tomography of the microgels at the air/water interface was investigated in Peak Force QNM mode, using an MSNL‐10‐E tip (Bruker) with a nominal resonance frequency of 38 kHz in air and a nominal spring constant of 0.1 Nm^‐1^ (radius: 2 nm; semi‐angle of the tip: 23 °; assumed sample Poisson's ratio: 0.3). Acquired data were processed using Nanoscope Analysis and a custom‐made MATLAB script.^[^ ^66^ ^]^

Confocal laser scanning microscopy (CLSM) measurements were carried out using an inverted confocal laser scanning microscope (Leica TCS SP5) equipped with a ×100 immersion objective with a numerical aperture of 1.4. A 543 nm HeNe laser was used to excite the red fluorescence of rhodamine B (543 nm). The CLSM was mounted in a thermostated enclosure (Life Imaging Services The Cube 2). Since the thermostated enclosure was not equipped with a cooling system, measurements were performed either at room temperature (23 °C) or using the thermostated enclosure at 31 °C. Concentrated core‐shell and hollow rod‐shaped microgel dispersions were prepared by concentrating approximately 500 μL of stock dispersions (0.59 wt.% for core‐shell microgels and 0.37 wt.% for hollow microgels) contained in an Eppendorf tube by centrifugation at 2000 rpm for 15 min. After removing most of the supernatant, the corresponding dispersion concentrations were estimated at 11.8 wt.% for core‐shell microgels and 8.8 wt.% for hollow microgels, respectively. The pellets were redispersed via vortexing and sonicated for 30 min, and the obtained dispersions (8 μL) were transferred to a confocal preparation where they were sandwiched between a glass slide and a coverslip separated by a watertight spacer that was 120 μm thick with a diameter of 51 mm (SecureSeal Imaging). The glass slide and coverslip were cleaned by ultrasonication in isopropanol for 15 min. In addition, the coverslip was ozone‐treated for 15 min using an UV Ozone Cleaner UVC‐104 (NanoBioAnalytics) to prevent adhesion of the microgels. The two preparations were equilibrated for 48 h at 20 °C in an incubator before imaging.

Static light scattering (SLS) measurements were performed using a goniometer from SLS‐Systemtechnik GmbH. This SLS setup was custom‐designed and extremely powerful for characterizing our systems. The combination of two different lasers (407 and 819 nm) allows to access an extended *q*‐range that was usually not accessible with standard commercially available instruments. The scattered intensities were measured from 15° to 150° in 1° steps. The measurements were conducted on 0.001 wt.% dispersions at temperatures ranging from 20 to 40 °C in increments of 4 °C. The sample temperature was controlled using an external water bath (Julabo). The scattering data were fitted using SasView software.

Dynamic light scattering (DLS) measurements were performed on an ALV setup connected to a goniometer and a Helium‐Neon (HeNe) laser with a wavelength of 633 nm. The DLS measurements were conducted on samples of silica rods, microgel‐coated silica rods, and hollow microgels (concentration: 0.001 wt.%) at temperatures ranging from 20 to 50 °C, measured in steps of 2 °C.

Fluorescence microscopy (FM) experiments were performed on a Nikon Eclipse TE300 inverted microscope equipped with a Nikon Plan Fluor oil objective (NA 0.5–1.3) and a magnification of 100x. Micrographs were recorded using a Blackfly S BFS‐U3‐04S2M camera for the hollow microgels and a Blackfly S BFS‐U3‐16S2M‐CS camera for the core‐shell microgels. Fluorescence imaging was conducted using a Nikon light source (an epi‐fluorescence illuminator, Nikon Intensilight) with fluorescent filters adapted for rhodamine, including a 561 nm laser (AHF Rhodamine Cy3 ET Filterset F46‐004, 545/25 ET bandpass, beamsplitter T 565 LPXR (25.5x36x1 mm^3^), and 605/70 ET bandpass). Interfacial experiments were performed using magnetically hermetically sealed cells as described in the Supporting Information at a temperature of 20 °C, maintained by a thermostated enclosure from Okolab equipped with both cooling and heating units.

Spinning disc confocal microscopy (SDCM) was performed for the characterization of the hollow microgel decorated emulsions on a Nikon Eclipse TI:HUBC/A inverted microscope body, fitted with a Nikon HP Plan Apo VC 100x/1.40 Oil objective, Yokogawa CSU‐X1FW spinning disk unit with a spinning frequency up to 5000 rpm. A beamsplitter Di01‐T405/488/568/647 quad band dichroic mirror. The micrographs were captured using an Andor iXon Ultra EMCCD (1024x1024 pixel, 26 fps, Electron Multiplying (EM) mode). For the excitation of the fluorescent dye at 561 nm a Cobolt Jive TEM 500 mW laser was used at 12 % power. The temperature was set at 25 °C using a thermostated chamber from Okolab mounted around the body of the microscope.

## Conflict of Interest

The authors declare no conflict of interest.

## Author Contributions

F. H. performed conceptualization, formal analysis, investigation, methodology, Writing ‐ original draft, Writing ‐ Review and editing. N.H. performed formal analysis, investigation, methodology, Writing ‐ Review and editing. V.D.L. performed formal analysis and investigation. A.J.A. performed formal analysis and investigation. M.F. performed formal analysis and investigation. A.B. performed formal analysis and investigation. O.‐V.L. performed formal analysis and investigation. D.B. performed formal analysis and investigation.

W.R. performed conceptualization, funding acquisition, project administration, resources, Writing ‐ Review and editing. J.J.C. performed conceptualization, formal analysis, investigation, methodology, funding acquisition, project administration, resources, supervision, investigation, Writing ‐ review and editing

## Conflict of Interest

The authors declare no conflict of interest.

## Supporting information

Supporting Information

Supplemental Video 1

Supplemental Video 2

Supplemental Video 3

Supplemental Video 4

Supplemental Video 5

## Data Availability

The data that support the findings of this study are available from the corresponding author upon reasonable request.
