# Supplementary material for: Soft and Deformable Thermoresponsive Hollow Rod‐Shaped Microgels
Source: Small. 2024 Sep 10;21(1):2401376. doi: 10.1002/smll.202401376 (PMC11707578; doi:10.1002/smll.202401376)
Supplement: Supplementary file 1 — Supporting Information [file SMLL-21-2401376-s001.pdf]

NANO · MICRO
small

Supporting Information

for *Small*, DOI 10.1002/smll.202401376

Soft and Deformable Thermoresponsive Hollow Rod-Shaped Microgels

*Fabian Hagemans, Nabanita Hazra, Viktoria D. Lovasz, Alexander J. Awad, Martin Frenken, Andrey Babenyshev, Olli-Ville Laukkanen, Dominik Braunmiller, Walter Richtering and Jérôme J. Crassous**

Supporting Information: Soft and Deformable Thermoresponsive Hollow Rod-Shaped Microgels

Fabian Hagemans, Nabanita Hazra, Viktoria D. Lovasz, Alexander J. Awad, Martin Frenken, Andrey Babenyshev, Olli-Ville Laukkanen, Dominik Braunmiller, Walter Richtering, and *Jérôme J. Crassous**

Institute of Physical Chemistry, RWTH Aachen University, Landoltweg 2, DE-52074 Aachen, Germany, European Union
Email Address: crassous@pc.rwth-aachen.de
Olli-Ville Laukkanen

VTT Technical Research Centre of Finland Ltd, Koivurannantie 1, 40400 Jyväskylä, Finland

The supporting information consists of seven sections. Section 1 presents a detailed description of the procedure followed for the synthesis of core-shell and hollow rod-shaped microgels. Section 2 is dedicated to the AFM characterization of the two systems. Supporting static light scattering measurements performed on the core-shell rod-shaped microgels are presented in Section 3. Section 4 focuses on the DLS analysis based on a spherocylinder model. Information and experiments related to interfacial assembly are provided in Section 5. A summary table with the different dimensions of the two systems determined by various methods is included in Section 6, and finally, Section 7 provides a description of the different supporting videos.

S1 Synthesis of the hollow rod-shaped microgels

The synthesis of the hollow rod-shaped microgels follows a four-step procedure outlined in detail in this section.

Step 1. The silica rods are prepared following a modified Stöber growth procedure. [1] First, fluorescently labeled silica seeds were prepared. Afterwards, 20 g of polyvinylpyrrolidone (PVP) was dissolved in 200 mL of 1-pentanol. 20 mL ethanol, 2 ml sodium citrate dihydrate (0.18 M), and 5 mL milli-Q water were added to this solution in a round flask. The mixture was then shaken by hand for 5 minutes. Subsequently, 4 mL of ammonia was added, and the solution was shaken again. In the final step, 2 mL of TEOS was added and after a brief stirring, the mixture was left to rest for four days, after which the excess PVP) was removed and the sample was transferred from 1-pentanol to absolute ethanol via centrifugation at 1500 g / 60 min. At this point, the sample is still polydisperse, however, by fractionation the width of the size distribution can be reduced significantly. Therefore, the larger rods were removed by centrifugation at 40 g for 15 min and collection of the supernatant. The smaller ones were removed by centrifugation at 800 g for 15 min and disposal of the supernatant. This process was continued until enough particles were collected. The dispersion was then stored in absolute ethanol on a rolling table to prevent aggregation of the silica rods until future applications.

Step 2. To ensure the presence of vinyl groups on the surface, the silica particles were modified with a thin layer of 3-(trimethoxysilyl)propyl methacrylate (TPM). First, the silica rods were transferred to 8.32 mL of absolute ethanol. The particles were sonicated for 30 minutes to remove all clusters. Then, 4.62 mL of ammonia (26.1 %) was added while stirring the solution. Over a 2-hour period, a total of 1574.0 mg of TPM was added and the solution was stirred for 3 hours to ensure complete coupling. To ensure complete condensation, the solution was heated to 75°C for one hour. After cooling down to room temperature, the particles were collected by centrifugation and redispersion in absolute ethanol (800 G, 25 min). Residual unreacted TPM was removed by two additional washing steps with absolute ethanol.

Step 3. A microgel shell is grown around the TPM-coated silica rods in a seeded polymerization reaction. First, the TPM-coated particles (160 mg) were transferred to water by centrifugation and redispersion. To

ensure the complete removal of traces of ethanol, the particles were washed one extra time with milli-Q grade water. Finally, the particles were dispersed in 8.1 mL milli-Q grade water and sonicated for 30 min to ensure complete redispersion. Then, in a three-necked round-bottomed flask, 2829.5 mg NIPAM (Sigma-Aldrich), 283.0 mg BIS (Sigma-Aldrich), and 45.0 mg KPS (Across Organics, 99 %) were dissolved in 192.0 mL milliQ grade water. After the addition of 2.80 mL dye solution (1.0 mg Rhodamine B methacrylate in 1.0 mL water*), the TPM-coated silica particles were added. While stirring, the mixture was purged with nitrogen for 60 min to ensure complete removal of oxygen. The polymerization of NIPAM was initiated by placing the flask in a pre-heated oil bath of 70°C. The mixture was stirred for 3 hours at 70°C. Before collecting the particles, the solution was allowed to cool down to room temperature while stirring slowly. Residual dye and secondary nucleation were removed by several centrifugation (800 G, 25 min) and redispersion steps, each time refreshing the supernatant with milliQ grade water. The samples were carefully redispersed using a roller table overnight. *To ensure complete dissolution of the rhodamine B methacrylate, the dye solution was prepared 24 hours in advance.

Step 4. The silica core is removed from the particles by mild etching in 0.1 M NaOH. As such, the batch of microgel coated silica rods was divided into two parts. One part was kept for further analysis. The other half was transferred to 50 mL 0.1 M NaOH and continuously mixed on a roller table for 4 days. To ensure complete removal of the silica template, the particles were transferred to fresh 0.1 M NaOH and mixed using a roller table for another 4 days. The hollow microgels were then redispersed in milliQ grade water by several centrifugation (800 G, 25 min) and redispersion steps, each time refreshing the supernatant with milliQ grade water. Complete removal of the template was confirmed by transmission electron microscopy.

We note that such synthetic approach can easily be scaled up to larger amount. The concentration of the systems is principally defined by the number density of the silica rods. Larger batch then essentially require to produce a larger amount of silica rods, which represent a significant effort in respect to the fractionation process. We refer to the work of Kuijk et al. for further details on the rod synthesis. [2, 3]

S2 Atomic force microscopy

AFM measurements were performed on a Dimension Icon AFM with a closed loop (Veeco Instruments Inc., software Nanoscope 9.4 (Bruker Corporation)). Measurement were conducted in a customized liquid cell on a heating stage (Dimension Icon Electrochemistry Chuck, Bruker Corporation) with temperature control (Model 335 Cryogenic Temperature Controller, Lake Shore Cryotronics). The microgels were measured at $T = 27$ °C, temperature was equilibrated for 60 min before measurement.
Contact stiffness measurements were carried out using the Peak Force QNM and Force Volume modes with modified MSNL-E (Bruker Corporation) tips. These E-tips have a nominal resonance frequency of 15 kHz in air and a nominal spring constant of 0.1 N/m, with a tip radius of 2 nm and a semi-angle of 23°. The AFM tip was activated by oxygen plasma treatment (PVA TePla plasma system 100) at 1.4 mbar oxygen pressure and 200W microwave power for 5 minutes immediately before measurements.

Initially, the Peak Force QNM mode was used to scan broad regions and identify areas of interest. Subsequently, areas containing one or two rods were selected for detailed measurements, with scan sizes of 5.0 x 5.0 µm^2 or 7.0 x 7.0 µm^2. Force Volume mode measurements were then performed with a 192 x 192 data point grid, a force threshold of 5 nN, a ramp size of 1.5 µm, a ramp rate of 10 Hz, and 3072 samples.

For analyzing the force volume data, a custom MATLAB routine was utilized [4]. In brief, all force-distance curves are baseline corrected. Then the contact point of each curve is defined, allowing the identification of part of the indentation and the correction of the height image. In the next step, the contact stiffness (the first derivative) of the force-indentation depth curves is determined. As sharp AFM probe is penetrating the porous polymer networks the local contact stiffness characterizes network density. Finally, the routine enables the visualization of the Force Volume data (Figure S1).

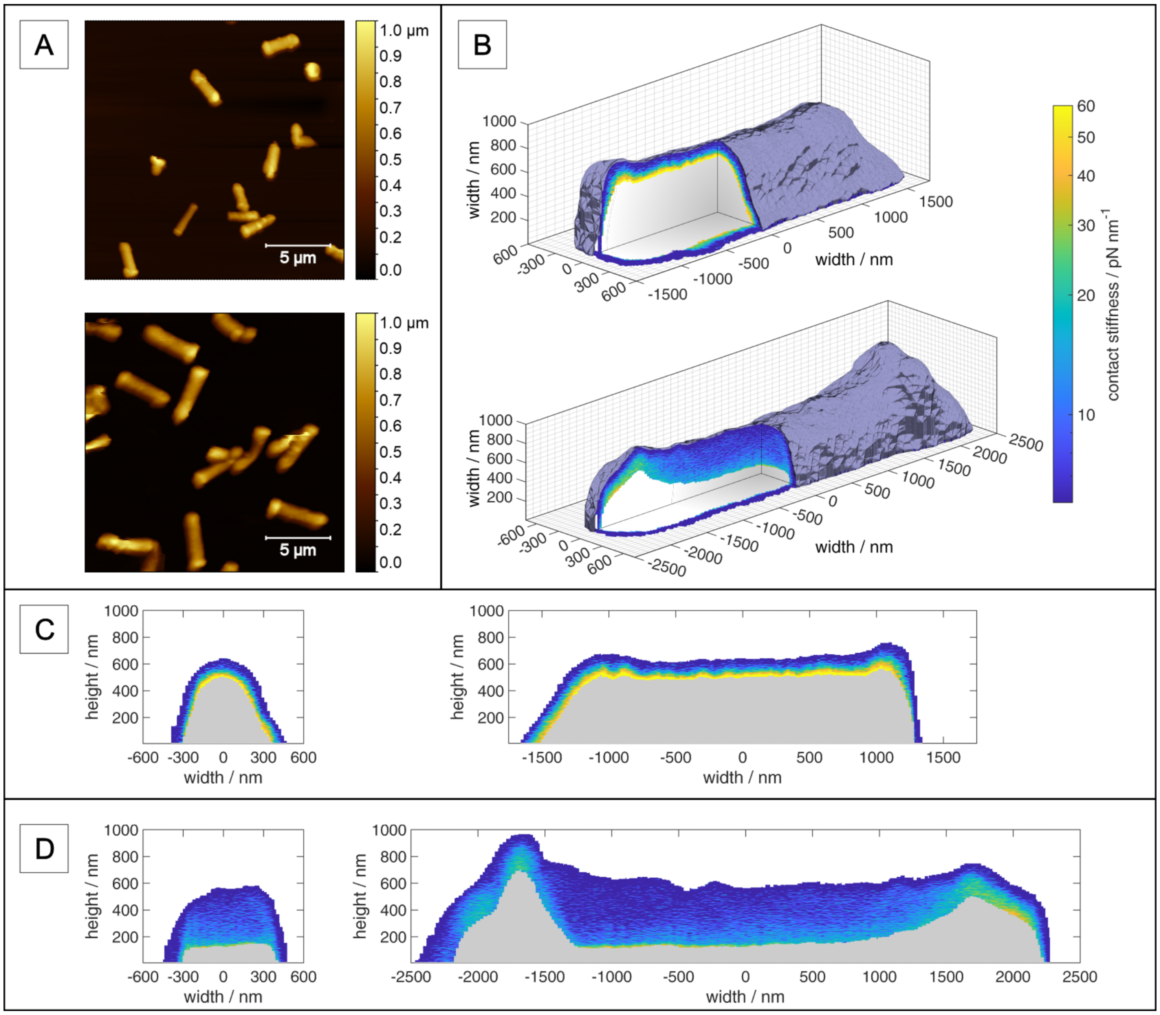

Figure S1: Representative AFM stiffness tomography of microgel coated silica rods (top) and etched hollow microgel rods (bottom) adsorbed on a glass slide. Height micrographs of microgels (20×20 μm^2) (A), cross-sections of the contact stiffness maps (B), profiles along the minor and major axes (C, D). Grey areas in the images indicate regions inaccessible to the AFM tip.

S3 Static light scattering

Supporting SLS measurements were performed on the core-shell rod-shaped microgels. Due to the large dimensions and large refractive index mismatch of the particles in water, SLS measurements were performed under matching conditions in a DMSO/water mixture of 97 vol% at 20 °C (Figure S2). The measurement was fitted with a core-shell cylinder model following a similar procedure as described for the hollow rods. This analysis led to a slightly lower average core width, determined to be 263 nm, compared to the TEM analysis of the silica core (313 nm), which could be related to the partial hydrolysis of the core. The average full width was determined to be 648 nm, which is in the same order but lower than that from the DLS under good solvent conditions (805 nm).

S4 Dynamic light scattering analysis

Data analysis

Dilute dispersions of core-shell and hollow rod-like microgels were measured by DLS (Goniometer ALV/CGS-5022F, ALV Gmbh, Langen, Germany) from 20 to 50°C with an increment of 2°C. The measurements were performed at different scattering angle θ from 30 to 90 ° every 10 °. DLS data were treated using the second-order cumulant analysis [5].

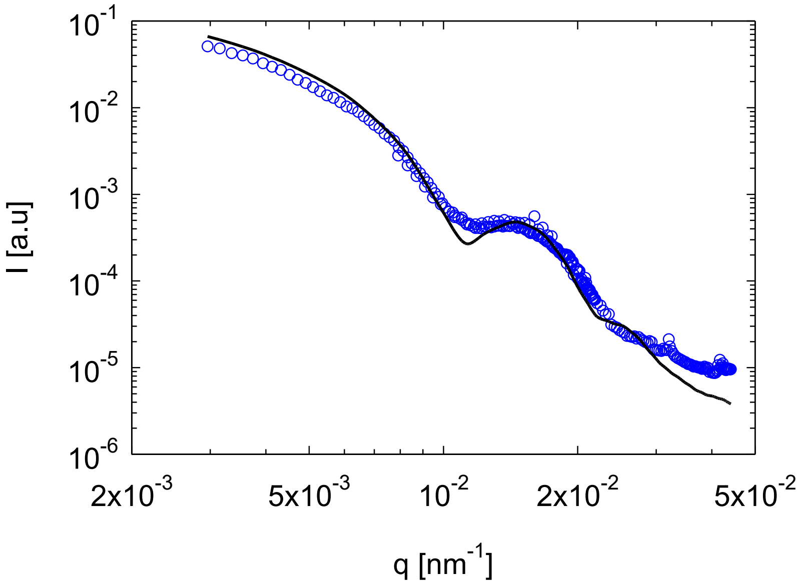

Figure S2: SLS measurement of the core-shell rod-shaped microgels in DMSO/water (97 vol%) (symbols) fitted with a core-shell cylinder model (line).

For monodisperse particles, the first-order electric field correlation function is a single exponential:

$$g^1(\tau) = exp(-\Gamma\tau) \tag{1}$$

with a decay rate of $\Gamma = D \cdot q^2$. D is the translational diffusion coefficient (in m^2 s^{-1}) of the particle and q is the scattering vector, with $q = (4\pi/\lambda)sin(\theta/2)$, that depends on θ, the refractive index of water n and the laser wavelength $\lambda = 632.8$ nm. For polydisperse particles, the second-order cumulant analysis allows writing g^1 as a deviation from a monoexponential:

$$g^1(\tau) = exp\left(-\bar{\Gamma}\tau + \frac{\mu_2}{2}\tau^2\right) \tag{2}$$

with $\bar{\Gamma}$ the average value of the decay rate and μ_2 the second order moment. The polydispersity can be defined as $\mu_2/\bar{\Gamma}$. In case of spherocylindrical particles, the translational diffusion coefficient is related to the translational friction coefficient f by the following equation:

$$D = \frac{k_B T}{f_{sp}} \tag{3}$$

With k_B the Boltzmann constant and T the temperature in Kelvin. For spherocylindrical particles, the friction coefficient, f_{sp}, is determined as follows [6, 7]:

$$f_{sp} = ln(\rho) + (3\pi\eta L) \,/ \left(\sum_{i=1}^{5} a_i \rho^{-i}\right) \tag{4}$$

Where ρ is the aspect ratio, L the length of the spherocylinder, η the solvent viscosity, and a_i the following constants: a_0=0.3863 , a_1=0.6863, a_2=0.0.06250, a_3=0.01042, a_4=0.000651 and a_5=0.0005859.

Determination of the swelling ratio and aspect ratio from the different model analysis.

The spherocylinder model described above was applied to estimate the temperature response of the long and short axes of the core-shell and hollow microgels from their diffusion coefficients. Different reference systems were considered, as described in the main manuscript, to extract these dimensions. Under constrained

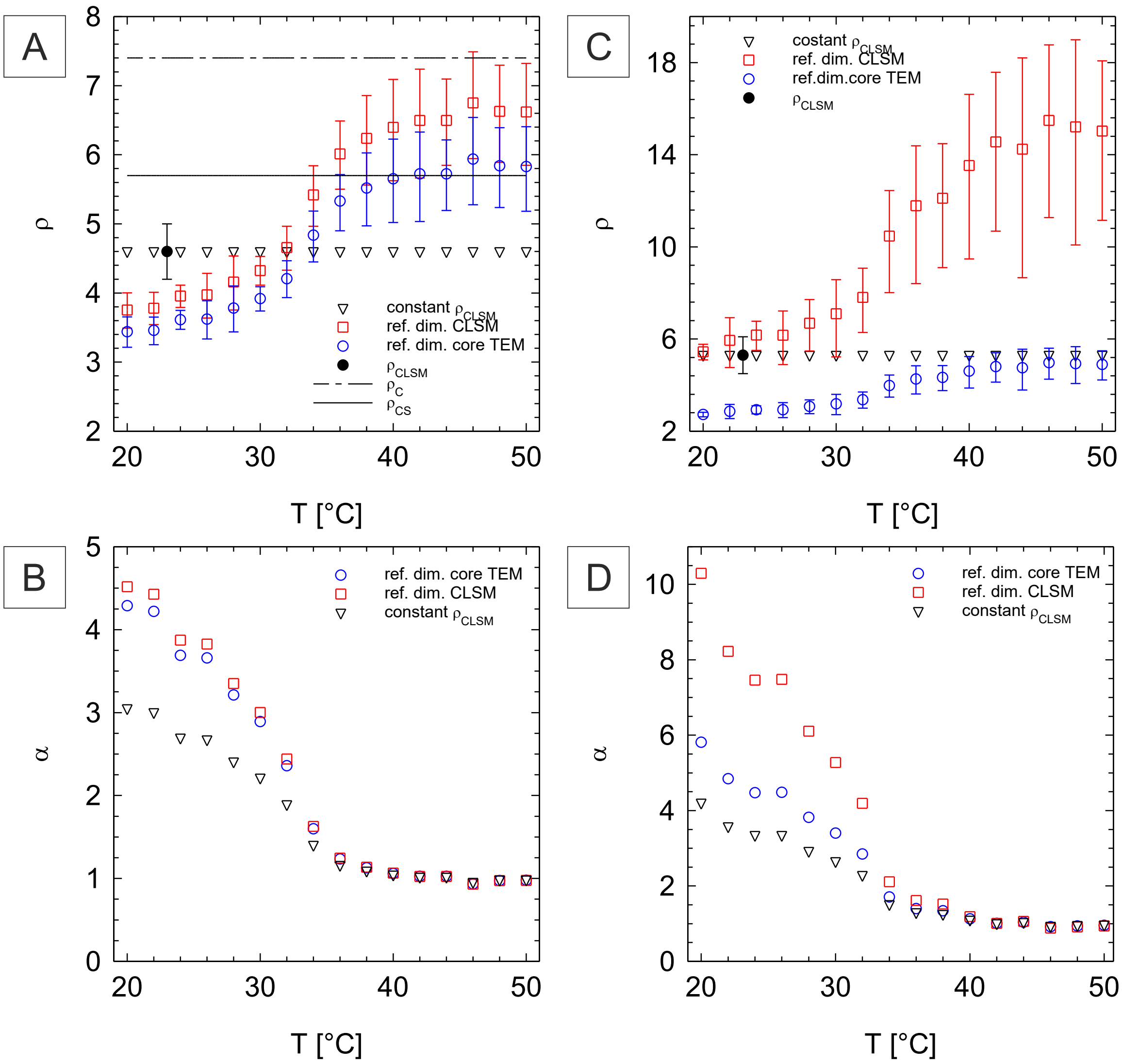

Figure S3: Aspect ratio, ρ, and swelling ratio, α, estimated from the different models for the core-shell (A,B) and hollow (C, D) rod-shaped microgels.

swelling conditions, the silica core dimensions are first considered as a reference. We assume a constant microgel thickness and determine the microgel thickness corresponding to different temperatures. This was then compared to a similar analysis using dimensions determined by CLSM as references, following a similar approach. Variations in the dimensions were assessed to reproduce the experimental data. Finally, in the third approach, we assume that the systems swell isotropically while maintaining their shape defined by a constant aspect ratio determined from CLSM measurements. A scaling factor is then derived to reproduce the data recorded at different temperatures. The aspect ratio ρ and swelling ratio α were determined from these calculations assuming a spherocylindrical shape and are summarized in Figure S3. Hereby, we clarify that α corresponds to the ratio of the total volume of the spherocylinder, $V(T)$, measured at temperature T, divided by the total volume of the spherocylinder averaged for $T \geq 40$ °C, $V_{collapsed}$ considering:

$$\alpha = \frac{V(T)}{V_{collapsed}} \tag{5}$$

with

$$V(T) = \frac{\pi D(T)^2 \left(3L(T) - D(T)\right)}{12} \tag{6}$$

and

$$V_{collapsed} = \langle V(T) \rangle_{T \geq 40^\circ C} \tag{7}$$

The aspect ratio and swelling value derived from the different models are summarized in Figure S3 for the core-shell (Fig. S3A,B) and hollow microgel systems (Fig. S3C,D).

S5 Interfacial assembly

Sample preparation for fluorescence microscopy at planar water/air interface

We designed magnetically hermetically sealed cells for imaging particles at the air-water interface, as shown in Figure S4. These cells were constructed by gluing a neodymium ring magnet with an outer diameter of 12 mm, an inner diameter of 9 mm, and a height of 1.5 mm onto a thin square glass coverslip measuring 22 × 22 mm^2. Photocurable epoxy glue was applied to the outer contact line of the magnet and the coverslip to prevent contamination from the glue during imaging. After curing the prepared open cell using a UV lamp and cleaning the assembly with isopropanol, the sample dispersion was filled into the magnet. It was then covered by a microscopic glass slide measuring 76 × 26 mm^2 with a thickness of 1.0 mm, and another loose ring magnet was placed on top to tightly seal the cell.

To investigate microgel assembly at the air-liquid interface at the single-particle level, the sample volume was adjusted between 28 and 45 µL. A convex layer is required to form an almost flat interface located about 30-70 µm from the coverslip. This allows for imaging particle assembly at the interface within the focus range of the employed 100X Plan Apo objective while compensating for evaporation and condensation of water. Since this preparation is hermetically sealed, due to the low volume of the cell, the atmosphere quickly saturates within it, allowing for investigation of the sample for several hours. Additionally, care must be taken to place and keep the solution horizontal to prevent disruption of the thin liquid film.

For preparing hollow microgel samples, stock dispersion was first vortex-mixed for 1 minute, then placed in hot water followed by a cold water bath, and finally sonicated for 3-5 minutes before checking under fluorescence microscopy to avoid aggregation. For dilution series, we prepared dilutions at factors of 5×, 10×, and 15× from stock solution following the same sample preparation process. Moreover, using closed magnetic cells, we investigated both stock solutions and their dilutions (5×, 10×, and 15×) at air-water interfaces via fluorescence microscopy. The experiments on core-shell microgels were performed on dispersions approximately at 0.1 wt%.

Adsorption of core-shell rod-shaped microgels at the air water interface

sedimentation of the rods and possibly lower surface activity. A concentration of approximately 0.1 wt% leads to the formation of large aggregates distributed across the entire interface. Similar to the hollow systems, the rods were also found to preferentially assemble flat at the interface. However, this assembly appears less defined than that of the hollow rods and seems to be affected by the polydispersity of the system, as well as by the presence of misshaped, partially hydrolyzed particles and a few aggregates, as shown in Figure S5. We also identified the formation of stacks where particles assemble side by side. By statistically analyzing the side-to-side distance over more than 100 configurations, we estimated the average side-to-side distance within these stacks to be $d_{SS} = 1229 \pm 115$ nm. This distance is significantly larger than the width of the particles determined from DLS analysis, attesting to their flattening at the interface. However, this value is significantly lower than that for hollow rods, which can deform more freely at the interface due to the absence of a solid silica core.

Emulsion preparation

To prepare the rod-shaped hollow microgel-Pickering emulsions, 10 μL decane was added in an eppendorf to 990 μL 0.01 $wt\%$ microgel dispersion. The mixture was then vortexed for 1 minute using a Scientific Industries Vortex-Genie 2.

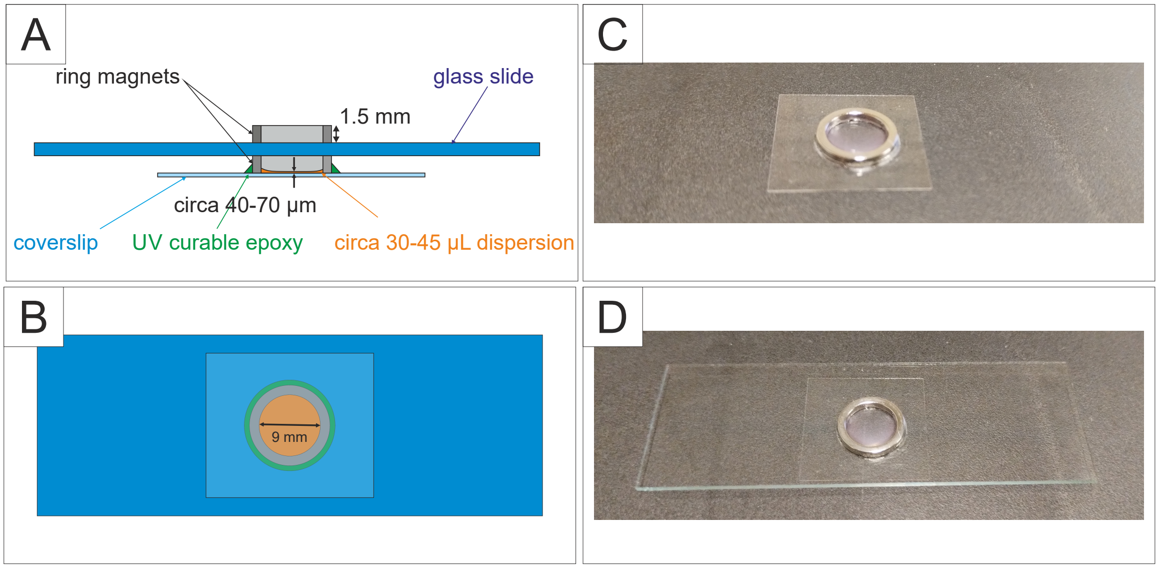

Figure S4: Schematic representation of the lateral cross-section (A) and top view (B) of the magnetically sealed closed cell for interfacial investigation. A photograph of the coverslip with a glued ring magnet containing the sample is shown in (C). The cell is then closed by placing a glass cover slide on top of the preparation and magnetically sealed with a second ring magnet on top of the slide as shown in (D). The whole preparation is then placed in an inverted fluorescent microscope. Due to the thin fluid film in the center of the preparation, the assembly of rod-shaped microgels can be imaged with a high magnification objective with a single particle resolution.

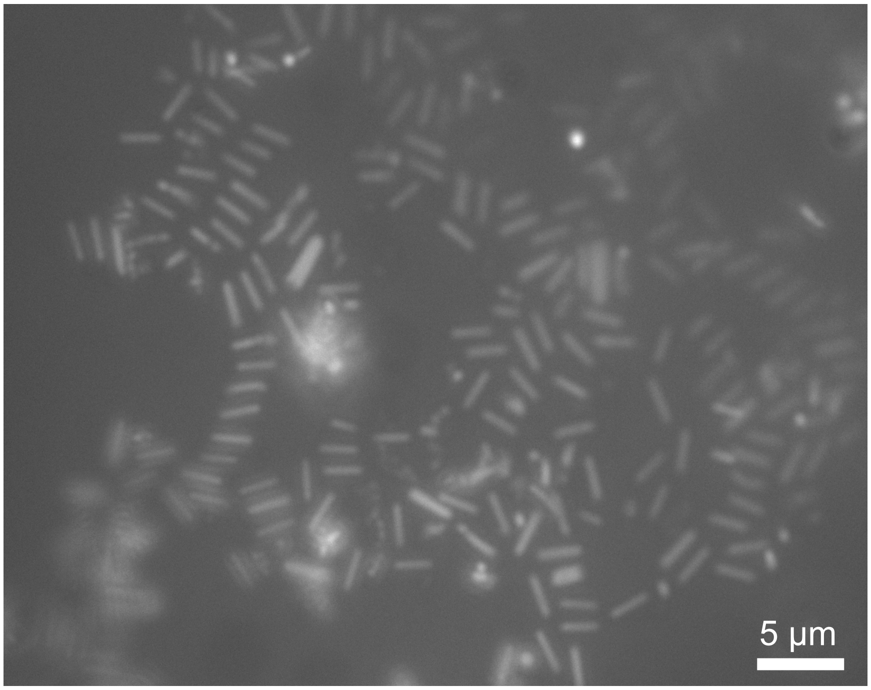

Figure S5: Fluorescence micrographs of the air-water interfacial assembly of the core-shell rod-shaped microgels measured at a concentration of circa of 0.1 wt%. Scale bars: 5 μm.

S6 Characterization of the rod-shaped microgel dimensions

	Core-shell rod-shaped microgels	Hollow rod-shaped microgels
L_{TEM} [nm]	2467 ± 236	5177 ± 555
D_{TEM} [nm]	436 ± 53	854 ± 116
ρ_{TEM}	5.7 ± 0.4	6.1 ± 0.8
$L_{CLSM}(23°C)$ [nm]	2697 ± 212	4233 ± 383
$D_{CLSM}(23°C)$ [nm]	587 ± 77	809 ± 147
$\rho_{CLSM}(23°C)$	4.6 ± 0.4	5.3 ± 0.8
$D_{T,SLS}(20°C)$ [nm]	648 ± 97 ¶	738 ± 111
$D_{C,SLS}(20°C)$ [nm]	263 ± 39 ¶	370 ± 56
$D_{SLS}(40°C)$ [nm]	-	562 ± 84
$D_{SLS}(40°C)$ [nm]	-	326 ± 49
$L_{DLS}(20°C)$ [nm]	2768 †	4127 ‡
$D_{DLS}(20°C)$ [nm]	805 †	789 ‡
$\rho_{DLS}(20°C)$	3.4 †	5.3 ‡
$L_{DLS}(40°C)$ [nm]	2385 †	2637‡
$D_{DLS}(40°C)$ [nm]	422 †	504 ‡
$\rho_{DLS}(40°C)$	5.6 †	5.2 ‡
$d_{SS}(20°C)$ [nm]	1229 ± 115	1612 ± 88
d_{SS}/D_{DLS}	1.5	2.0

¶ Measurements under silica index-matching conditions in 97 vol% DMSO/water mixture. † Analysis performed using the dimensions of the silica core as reference. ‡ Analysis performed considering a constant aspect ratio from the CLSM analysis.

Table S1: Summary of the dimensions of the core-shell and hollow rod-shaped microgels.

S7 Supporting Videos

Video S1: Dynamics of a concentrated core-shell rod-shaped microgel dispersion measured at the surface of the glass coverslip by CLSM at 23 °C.
Video S2: Dynamics of a concentrated hollow rod-shaped microgel dispersion measured at the surface of the glass coverslip by CLSM at 23 °C.
Video S3: Z-stack of a concentrated core-shell rod-shaped microgel dispersion measured from the surface of the glass coverslip to the bulk by CLSM at 23 °C.
Video S4: Z-stack of a concentrated hollow rod-shaped microgel dispersion measured from the surface of the glass coverslip to the bulk by CLSM at 23 °C.
Video S5: Dynamics of a concentrated hollow rod-shaped microgel dispersion measured at the surface of the glass coverslip by CLSM at 31 °C.

References

[1] W. Stöber, A. Fink, E. Bohn, *Journal of colloid and interface science* **1968**, *26*, 1 62.

[2] A. Kuijk, A. van Blaaderen, A. S. o. M. Imhof, *J. Am. Chem. Soc* **2011**, *133* 2346.

[3] A. Kuijk, D. V. Byelov, A. V. Petukhov, A. van Blaaderen, A. Imhof, *Faraday Discuss.* **2012**, *159* 181.

[4] M. F. Schulte, S. Bochenek, M. Brugnoni, A. Scotti, A. Mourran, W. Richtering, *Angewandte Chemie International Edition* **2021**, *60*, 5 2280.

[5] R. Pecora, *Dynamic light scattering: applications of photon correlation spectroscopy*, Springer Science & Business Media, **1985**.

[6] V. N. Tsvetkov, *Rigid-Chain Polymers: Hydrodynamic and Optical Properties in Solution*, Consultants Bureau (Plenum), New York, **1989**.

[7] I. Martchenko, H. Dietsch, C. Moitzi, P. Schurtenberger, *J. Phys. Chem. B* **2011**, *115*, 49 14838.
